# Influence of Preservation of Normal Knee Contact Stress on Other Compartments with respect to the Tibial Insert Design for Unicompartmental Knee Arthroplasty

**DOI:** 10.1155/2019/9246379

**Published:** 2019-11-14

**Authors:** Yong-Gon Koh, Kyoung-Mi Park, Kyoung-Tak Kang

**Affiliations:** ^1^Joint Reconstruction Center, Department of Orthopaedic Surgery, Yonsei Sarang Hospital, 10 Hyoryeong-ro, Seocho-gu, Seoul 06698, Republic of Korea; ^2^Department of Mechanical Engineering, Yonsei University, 50 Yonsei-ro, Seodaemun-gu, Seoul 03722, Republic of Korea

## Abstract

Recent advances in imaging technology and additive manufacturing have led to the introduction of customized unicompartmental knee arthroplasty (UKA) that can potentially improve functional performance due to customized geometries, including customized sagittal and coronal curvature and enhanced bone preservation. The purpose of this study involved evaluating the biomechanical effect of the tibial insert design on the customized medial UKA using computer simulations. We developed sagittal and coronal curvatures in a native knee mimetic femoral component design. We utilized three types of tibial insert design: flat, anatomy mimetic, and conforming design. We evaluated contact stress on the tibial insert and other compartments, including the lateral meniscus and articular cartilage, under gait and squat loading conditions. For the conforming UKA design, the tibial insert and lateral meniscus exhibited the lowest contact stress under stance phase gait cycle. However, for the conforming UKA design, the tibial insert and lateral meniscus exhibited the highest contact stress under swing phase gait cycle. For the flat UKA design, the articular cartilage exhibited the lowest contact stress under gait and squat loading conditions. The anatomy mimetic UKA design exhibited the most normal-like contact stress on the other compartments under gait and squat loading conditions. The results reveal the importance of conformity between the femoral component and the tibial insert in the customized UKA. Based on the results on the femoral component as well as the tibial insert in the customized UKA, the anatomy mimetic design preserves normal knee joint biomechanics and thus may prevent progressive osteoarthritis of the other knee compartments.

## 1. Introduction

Osteoarthritis (OA) typically first affects the medial compartment of the tibiofemoral (TF) joint [1] and is a growing concern in younger patients [2]. There are various surgical treatments for isolated medial compartment arthritis, including unicompartmental knee arthroplasty (UKA), total knee arthroplasty (TKA), and high tibial osteotomy [3]. The utilization rate of UKA exhibits a growth rate three times than that of TKA. Outstanding and dependable clinical results in the first decade of its use led surgeons to expand the indication for UKA to younger and more active patients [4]. The advantages include a faster recovery rate due to minimally invasive surgery, less bone loss, better functional outcomes, and lower complication rates [5]. However, UKA involves a demanding surgical technique, and precise component positioning is essential [6].

Although patient factors play a role in UKA survivorship, current UKA designs present an important limitation [7]. Various anatomical studies indicate a wide range of variability in the size and shape of the medial and lateral tibial components [8, 9]. High early failure rates are reported in obese patients for designs with an inset or narrow tibia, while early results for wider tibial components exhibit lower early failure rates [10, 11]. Asians exhibit a smaller build and stature when compared to their Western counterparts. However, most prostheses currently available in the market are produced to fit the physique of Caucasian patients [12]. The aforementioned difference was also observed in terms of sex, in addition to ethnicity [13, 14]. To solve the problem, patient-specific or customized implants are developed and introduced [15]. A customized UKA can provide superior cortical bone coverage and fit with minimal overhang and undercoverage compared to off-the-shelf UKA [16]. Additionally, a recent computer simulation study indicates that a customized UKA can yield mechanics closer to that of a healthy knee joint [17].

A potential disadvantage of a completely customized UKA is variability in the coronal and sagittal curvature of the femoral component, which results in point loading at select flexion angles when a curved tibial insert is used [18]. To address this problem, a flat polyethylene (PE) tibial insert is paired with a constant coronal curvature femoral component, and this guarantees constant loading conditions over a large area, irrespective of the flexion angle [15, 17, 18]. However, this type of flat design involves a problem that does not describe tibial insert anatomy.

The aim of this study involved evaluating the biomechanics of different tibial insert conformity designs to provide a design that is closer to that of a healthy knee joint. Thus, we developed three different tibial insert surface designs: flat, anatomy mimetic, and conforming tibial insert customized UKAs. We hypothesize that the anatomy mimetic customized UKA provides biomechanics closer to that of the healthy knee joint.

## 2. Materials and Methods

### 2.1. Design of Customized UKA

**Figure 1:**
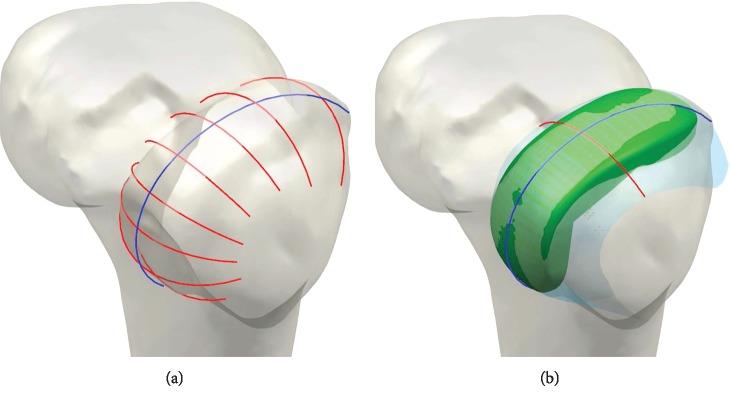
(a) Intersection curves were used to extract the articulating surface geometry in the sagittal and coronal planes and (b) in the development of the femoral component of the patient-specific UKA using sagittal curves and constant coronal curves.

The customized medial UKA was designed by using a preexisting three-dimensional (3D) knee joint model [17, 19–21]. The customized medial UKA design was initiated with the acquisition of medical image data. Planes were introduced using the intersection of condyles in both sagittal and coronal views. Intersection curves were used to extract the articulating surface geometry in both planes, which were imported into Unigraphics NX (version 7.0; Siemens PLM Software, Torrance, CA, USA) and fitted with rational B splines (Figure 1(a)) [17, 18, 21–23].

The patient's bone defines the sagittal geometry of the femoral component. Thus, the sagittal geometry is completely patient-specific, and the resultant sagittal implant radii vary along the anteroposterior dimension of the implant [17, 18, 21–23]. The coronal curvatures of the patient are measured at multiple positions along the length of the femoral condyle. An average curvature is then derived for each patient. Using this approach, a patient-derived constant coronal curvature is achieved (Figure 1(b)). The tibial component is designed based on the CT and MRI data of the patient's tibia to ensure complete cortical rim coverage. With this method, the patient receives an implant with an optimized fit. The tibial plateau and inserts are designed for minimal bone cut and provide a smooth articulating surface for the femoral component. The tibial component is patient-specific, and thus, it can potentially provide complete cortical rim coverage, which cannot be achieved with a conventional implant [24].

**Figure 2:**
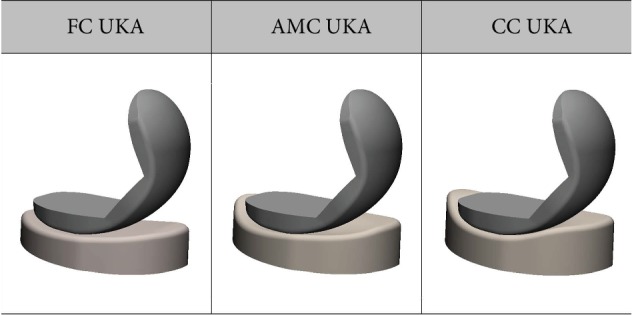
Cross-sections of the femoral component and tibial insert of the customized UKA used in this study, with three different conformities.

We designed three different tibial insert conformities (Figure 2). Generally, the flat design is used for the tibial insert in a fixed-bearing UKA [25], which is similar to a customized fixed-bearing UKA. Additionally, the customized design exhibits variability in the coronal curvature of the femoral component and results in point loading at select flexion angles when a curved tibial insert is used [17, 18]. To address that problem, a flat tibial insert is paired with a constant coronal curvature femoral component, and this provides constant loading conditions over a large area, irrespective of the flexion angle [17, 18]. Therefore, we developed tibial insert conformity in flat customized (FC) UKA as the initial design. For the second design, the real medial geometry was measured, and a medial anatomy mimetic customized (AMC) UKA was developed. The sagittal cross-section of the medial tibial insert has a concave geometry similar to that of the native medial tibial cartilage, including a shallow curvature for overcoming the stability provided by the missing meniscus. As previously mentioned, the femoral component coronal curvature varies, and edge loading may occur in the conforming design. However, the implant is used in the customized UKA, and various tibial insert designs can be applied. Therefore, the third design corresponds to a conforming customized (CC) UKA. Additionally, the femoral component designs were identical in the customized UKA.

### 2.2. Finite Element Model

**Figure 3:**
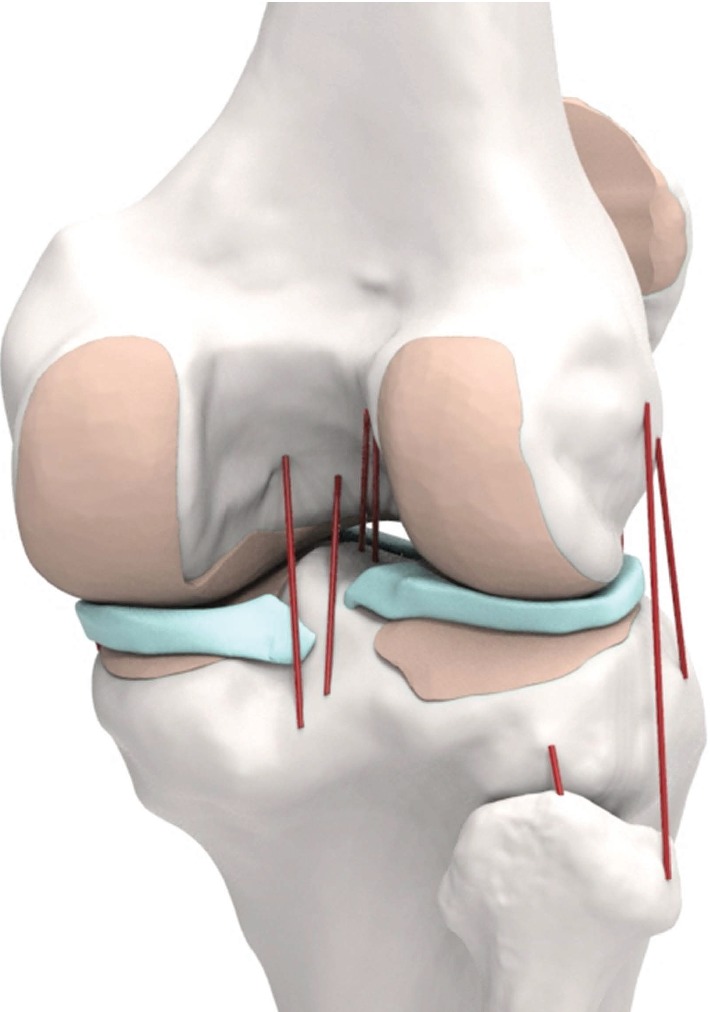
Validated FE native knee model used in this study, including TF and PF joints and major ligaments.

The 3D medical imaging data used for the customized UKA design were also used in the development of the finite element (FE) model [17, 19, 20]. The intact knee joint model had previously been developed and validated [17, 19, 20], and the procedure can be found in the literature. The FE model comprises the TF and patellofemoral (PF) joints and major ligaments (Figure 3).

All ligament bundles were modeled as nonlinear springs, and the material properties were obtained from a previous study [26]. The ligament insertion points were set with respect to the anatomy obtained from magnetic resonance imaging sets of the subject. The description is available in previous studies [27, 28]. The ligaments were simulated as nonlinear force elements, and their parabolic and linear equations are as follows: if *ε* < 0, *f*(*ε*) = 0; if 0 ≤ *ε* ≤ 2*ε*_1_, *f*(*ε*) = *kε*^2^/4*ε*_1_; and if *ε* > 2*ε*_1_, *f*(*ε*) = *k*(*ε* − *ε*_1_), where *f* denotes the tension of the ligament, *ε* denotes the ligament strain, and *k* is the stiffness coefficient of each ligament. The linear range threshold was specified as *ε*_1_ = 0.03. In all test scenarios applied in this study, the soft tissue elements remained in the same position. The bony structures were modeled as rigid bodies using four-node shell elements [29] while the interfaces between the articular cartilage and the bones were modeled as fully bonded [29]. Six pairs of tibiofemoral contact between the femoral cartilage and the meniscus, the meniscus and the tibial cartilage, and the femoral cartilage and the tibial cartilage were modeled for both the medial and lateral sides of the joint [17]. The heights of the tibial insert for the three different designs were matched to the original bone anatomy using a neutral mechanical alignment, cutting the tibia orthogonal to the coronal tibial mechanical axis [17]. The rotating axis was defined as the line parallel to the lateral edge of the tibial baseplate passing the center of the femoral component fixation peg. For the implanted model, a 1 mm cement gap was simulated between the component and the bone. The materials of the femoral component, PE insert, tibial baseplate, and bone cement included cobalt-chromium-molybdenum (CoCrMo) alloy, ultrahigh-molecular-weight polyethylene (UHMWPE), titanium alloy (Ti6Al4V), and polymethyl methacrylate (PMMA), respectively (Table 1) [17, 20, 30]. The femoral component requires contact with the tibial insert, and the coefficient of friction between the PE and the femoral component was selected as 0.04 [30].

The FE simulation comprised three types of loading conditions corresponding to the loads used in the experiment for model validation and the prediction of daily activity loading scenarios. For the first loading condition, 150 N was applied to the tibia at 30° and 90° flexion in the FE knee joint to measure anterior-posterior (AP) tibial translations [19]. Furthermore, a second axial loading of 1,150 N was applied to the model to obtain contact stresses, which were compared to those reported in a published study on the FE knee joint model [29]. The third loading condition, which corresponds to the gait cycle, and squat loading conditions, was applied to evaluate knee joint mechanics. Computational analysis was conducted by applying an AP force to the femur with respect to the compressive load applied to the hip, with constrained femoral internal-external rotation, free medial-lateral translation, and knee flexion determined through a combination of the vertical hip and the load of the quadriceps. Thus, a six-degree-of-freedom TF joint was created [31–33]. A proportional-integral-derivative controller was incorporated into the computational model to control the quadriceps in a manner similar to that in a previous experiment [34]. A control system was used to calculate the instantaneous displacement of the quadriceps muscle, and this was required to match the same target flexion profile used in the experiment. Internal-external and varus-valgus torques were applied to the tibia while the remaining tibial degrees-of-freedom were constrained [31–33].

The FE model was analyzed using ABAQUS software (version 6.11; Simulia, Providence, RI, USA). The study investigated and compared the contact stress on the PE insert and other compartments of the customized UKA designs with three different conformities to a native knee. The kinematics were calculated based on Grood and Suntay's definition of a joint coordinate system [35].

## 3. Results

### 3.1. Intact Model Validation

The intact FE model was compared to the experiment using the Fe model's subject for validation purposes. Under the loading condition with a 30° flexion, the anterior tibial translation was 2.83 mm in the experiment and 2.54 mm in the FE model, and the posterior tibial translation was 2.12 mm in the experiment and 2.18 mm in the FE model. At 90° flexion, the anterior tibial translation was 3.32 mm in the experiment and 3.09 mm in the FE model, and the posterior tibial translation was 2.64 mm in the experiment and 2.71 mm in the FE model. The experimental results show good agreement with those obtained using the FE model [19]. Furthermore, the intact FE model was validated by comparing it with computational results from previous studies. Under an axial load of 1,150 N, average contact stresses of 3.1 MPa and 1.53 MPa were observed on the medial and lateral menisci, respectively. Both are within 6% of the 2.9 MPa and 1.45 MPa contact stress values reported by Pena et al. [29]. These minor differences may be due to geometrical variations between the different studies, such as the thickness of the cartilage and meniscus. The significant consistency between the validation results and the results reported in extant studies is indicative of the validity of the results obtained with the FE model in this study.

### 3.2. Comparison of the Contact Stress on the PE Insert and Other Compartments of the Customized UKA Designs with Three Different Conformities against That on a Native Knee under Gait Cycle and Squat Loading Conditions

**Figure 4:**
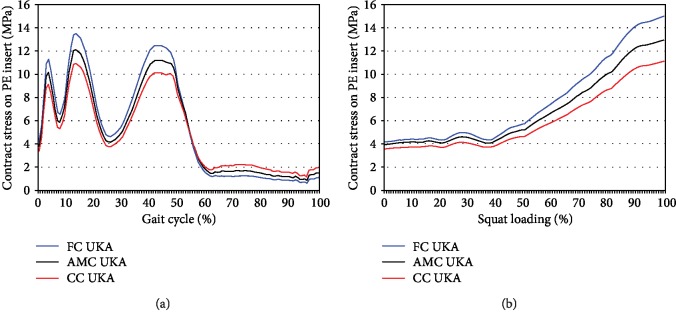
Comparison of the contact stress on the PE insert of three customized UKA designs with three different conformities under (a) gait and (b) squat loading conditions.

Figure 4 shows the contact stress on the PE insert of the three different tibial insert designs for the customized UKA under gait and squat loading conditions. During the stance phase gait cycle, a difference was observed in the PE insert contact stress of the three different tibial insert designs for the customized UKA. The same trend was also observed under the squat loading conditions. CC UKA exhibited the lowest PE inset contact stress under stance phase gait cycle, followed by AMC UKA and FC UKA. Under the squat loading conditions, CC UKA exhibited the lowest PE insert contact stress. During the swing phase, CC UKA exhibited the highest PE inset contact stress, followed by AMC UKA and FC UKA.

**Figure 5:**
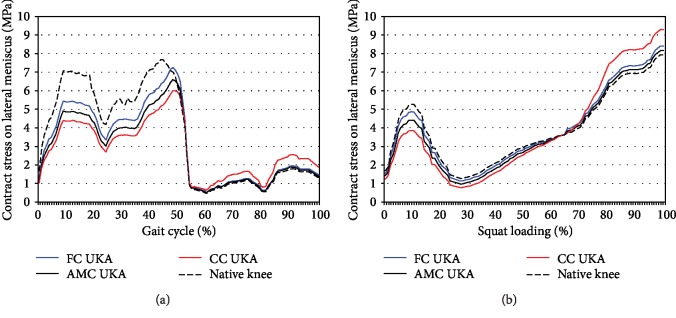
Comparison of the contact stress on the lateral meniscus in three customized UKA designs with three different conformities against that on a native knee under (a) gait and (b) squat loading conditions.

Figure 5 shows the contact stress on the lateral meniscus for different tibial insert designs and a native knee joint under gait and squat loading conditions. Contact stress on the lateral meniscus in the native knee was higher than that in the three different tibial insert designs for the customized UKA during the stance phase gait cycle. The trend of contact stress on the lateral meniscus was also observed under deep flexion squat loading conditions. The lateral meniscus, like the PE insert, exhibited high contact stress during the stance phase and low contact stress during the swing phase for the three different tibial insert designs for the customized UKA, compared to the native knee.

**Figure 6:**
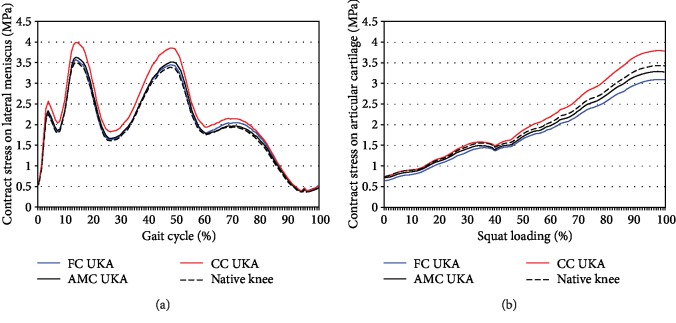
Comparison of the contact stress on the articular cartilage in three customized UKA designs with three different conformities against that on a native knee under (a) gait and (b) squat loading conditions.

Figure 6 shows the contact stress on the articular cartilage for the three different tibial insert designs for the customized UKA under gait and squat loading conditions. During the gait cycle, contact stress on the articular cartilage in the native knee was lower than that in the three different tibial insert designs for the customized UKA. FC UKA and CC UKA exhibited higher contact stress on the articular cartilage than on the native knee in the swing phase. Furthermore, the CC UKA exhibited higher contact stress on the articular cartilage than on the native knee under high flexion squat loading conditions. Under gait and squat loading conditions, the contact stress on the lateral meniscus and articular cartilage indicates that the AMC UKA is closest to normal contact mechanics.

## 4. Discussion

The most important finding of this study is that the AMC UKA exhibits close to native knee contact mechanics. Therefore, the AMC UKA prevents progressive OA of other compartments.

We evaluated contact stress, which is closely related to degenerative OA of the knee joint after medial UKA [36, 37]. A previous study indicates that after a minimum follow-up duration of ten years, medial UKA is associated with excellent clinical and radiographic results [38]. Although the ten-year survival rate is excellent, radiographic signs of progression of OA were observed in the other compartments [38]. Theoretically, UKA requires a technically demanding procedure and precise component positioning [6, 39]. Furthermore, UKA entails challenges due to surgical difficulties, such as device failures, residual pain, subsidence, and OA progression in the other compartments [38, 40]. To overcome this problem, a customized instrumentation technique is applied to UKA.

Bell et al. evaluated the accuracy and clinical outcomes of the customized instrumentation implementation technique using a fixed-bearing UKA [41]. They proved that the technique might offer specific advantages to surgeons who perform lower volumes of UKA and can potentially improve both clinical outcomes and implant survivorship of UKA and achieve greater consistency in results [41]. However, it is not possible for this type of customized instrumentation to resolve the effect of morphology with respect to ethnicity and gender differences. The Asian population exhibits a smaller build and stature compared to the Western population [12]. A majority of conventional UKA prostheses are designed to match the Caucasian physique [42]. In UKA, the geometry of the femoral and tibial components should match the resected surface to the maximum extent possible to provide optimal stability and load transfer [42]. Koeck et al. indicated that customized instrumentation and implant using fixed-bearing UKA can reliably restore the leg axis, obtain a medial proximal tibial angle of 90°, prevent implant malpositioning, and ensure maximal tibial coverage [43]. Steklov et al. indicated that a constant coronal curvature can be applied to a customized UKA by measuring coronal curvatures across the femoral condyle in each patient and by deriving the average curvature [18]. This novel approach combines the unique benefits of customized geometry with proven design concepts in UKA to minimize PE wear [18]. However, as previously mentioned, the customized UKA should overcome edge loading at select flexion angles when a curved tibial insert is used [17]. To address the problem, a flat PE tibial insert is paired with the constant coronal curvature femoral component, and this ensures constant loading conditions over a large area, irrespective of the flexion angle [17, 18]. However, in a native knee, the medial and lateral tibial plateaus exhibit anatomical asymmetric geometries with a slightly dished medial plateau and a convex lateral plateau.

The result presents the pattern of various contact stresses on the PE tibial insert and other compartments in the customized UKA with respect to different tibial insert designs. An interesting finding was observed in CC UKA: the CC UKA exhibited increased contact stress on the PE insert during the swing phase gait cycle and high flexion during squat loading conditions. The most influential factor on contact stress is the contact area. Therefore, the CC UKA with an increased contact area should exhibit decreased contact stress, although it did not exhibit this. Generally, conforming design is used in the mobile-bearing UKA [36]. However, in this study, the conforming design was used in the fixed-bearing UKA. Abnormal kinematics and increased contact stress were observed, and this was similar for the swing phase and high flexion. When flexion increased, for the CC UKA, movement of the tibial insert restores a similar contact area. However, edge loading may occur in a fixed condition. For the stance phase gait and deep flexion under squat loading conditions in which the flexion angle does not show a significant effect, the CC UKA exhibited the lowest contact stress due to the advantage of conformity.

In the lateral meniscus, a trend of contact stress similar to that in the PE insert was observed in the customized UKA for the three different tibial insert designs. This trend is probably due to the role the menisci play in protecting the TF cartilage layers when the load is transferred. When the UKA was implanted, the contact stress on the lateral meniscus is lower than that in the native knee during the stance phase of the gait cycle in which loading is mainly involved. This is primarily due to the change in stiffness between the medial and lateral compartments induced in the knee by the device [44].

On the lateral side, the cartilage layer of the TF exhibits an elastic modulus of 15 MPa. In contrast to the cartilage layers, the tibial articular insert exhibits an elastic modulus of 685 MPa. Consequently, the material characteristics of the medial and lateral compartments differ by more than 40 times. Notably, other compartments in the AMC UKA have the advantage of contact mechanics similar to that of the native knee in swing phase gait and high flexion. CC UKA and FC UKA showed kinematic change, which led to lateral cartilage contact stress because they did not restore tibial insert conformity and native anatomy. This trend was found for swing phase gait and high flexion squat loading conditions. The most important advantage of the AMC UKA was observed under high flexion where the effect of the anatomy mimetic tibial insert was visible as the *J* curve of the femur was maintained in the femoral component.

The contact area is most important during the stance phase gait cycle and deep flexion during squat loading conditions, during which the axial force was primarily visible. However, the contact area, as well as the kinematics, is also crucial during the swing phase gait cycle and high flexion under squat loading conditions. Unfortunately, both the femur and tibial mimetic AMC UKAs could not preserve perfect normal knee contact mechanism. An important factor is that change in the mechanism due to change in material stiffness plays the most crucial role, even if it corresponds to an anatomy mimetic design. Furthermore, the tibial insert could not perfectly replicate the role of mobile meniscus characteristics. Generally, there are significant differences between the biomechanics of the medial and lateral menisci [45, 46]. The medial meniscus is significantly less mobile than the lateral meniscus due to its attachment to the medial collateral ligament and larger insertion areas.

In terms of clinical relevance, it is not possible to apply a conforming design to the tibial insert when a customized UKA is developed. Bernasek et al. reported unsatisfactory results regarding the insertion of the same type of conforming fixed-bearing UKA [47]. Furthermore, a previous study indicated that significant degenerative changes in the other compartments occurred in only one of the eighty-seven knees in which an unconstrained UKA was implanted [48]. The results support the reliability of this study. The AMC UKA should apply mobile characteristics to the tibial insert to preserve knee mechanics closer to that of the native knee. However, a reason for the application of the conforming design to mobile-bearing UKA involves preventing bearing dislocation. Therefore, a spinout mechanism should be considered for preventing dislocation through the application of mobile characteristics in the AMC UKA to preserve native knee mechanics.

Two strengths of this study are as follows: First, unlike previous UKA studies, the FE model included the tibia, femur, and related soft tissues [49, 50]. Second, unlike the current biomechanical UKA model, this study included the application of gait and squat loading conditions [49, 50].

Nevertheless, several limitations should also be noted. First, the bony structures were assumed as rigid, while in reality, bone exhibits cortical and cancellous tissues. However, the primary purpose of the study did not involve evaluating the effects of different prostheses on bone. Furthermore, the assumption exerted minimal influence on the study because the stiffness of bone exceeds that of the relevant soft tissues [29]. Second, the computational model represented a customized UKA and the results are not necessarily expected to extend to other implant designs, such as the customized mobile-bearing UKA. Third, the material properties and attachment points of the ligaments were assumed in the model based on values from extant studies, although significant variability exists regarding reported values. However, the objective did not involve determining the actual values of ligament forces but determining the effect of variability in a customized fixed-bearing UKA with respect to the tibial insert design corresponding to the femoral component. Furthermore, the advantage of computer simulation of a single subject is that we could determine the effects of the tibial insert design of a customized UKA within the same *individual* and eliminate the effects of other variables, such as weight, height, bony geometry, ligament properties, and component size [51].

## 5. Conclusion

The anatomy mimetic design, which retains the native tibial insert, exhibited significant contact mechanics improvement over the customized UKA during gait and squat loading conditions. The nonanatomic tibial insert geometry of the customized UKA contributed to contact mechanics abnormalities, including the PE tibial insert and the other compartments. Therefore, the AMC UKA may represent an essential step in our attempt to restore the function of the native mechanics of the knee. Based on the results for the femoral component as well as the tibial insert in a customized UKA, the anatomy mimetic design preserves normal knee biomechanics and thus may prevent progressive osteoarthritis of the other compartments.

## Figures and Tables

**Table 1:** Material properties of the FE model.

	Young's modulus (MPa)	Poisson's ratio
CoCrMo alloy	220,000	0.30
UHMWPE	685	0.47
Ti6Al4V alloy	110,000	0.30
PMMA	1,940	0.40

## Data Availability

The data used to support the findings of this study are included within the article.
